# Reporting frequency of radiology findings increases after introducing visual rating scales in the primary care diagnostic work up of subjective and mild cognitive impairment

**DOI:** 10.1007/s00330-020-07180-2

**Published:** 2020-08-26

**Authors:** Claes Håkansson, Gustav Torisson, Elisabet Londos, Oskar Hansson, Isabella M. Björkman-Burtscher, Danielle van Westen

**Affiliations:** 1 Department of Imaging and Function, Skånes University Hospital, Lund, Sweden; 2 Department of Clinical Sciences, Diagnostic Radiology, Lund University, Lund, Sweden; 3 Department of Translational Medicine, Clinical Infection Medicine, Lund University, Malmö, Sweden; 4 Department of Clinical Sciences, Clinical Memory Research Unit, Lund University, Lund, Sweden; 5 Memory Clinic, Skåne University Hospital, Malmö, Sweden; 6 Department of Radiology, Clinical Sciences, Sahlgrenska Academy, University of Gothenburg, Gothenburg, Sweden

**Keywords:** Brain, Dementia, Tomography X-ray computed

## Abstract

**Objectives:**

Study the effect of introducing a template for radiological reporting of non-enhanced computed tomography (NECT) in the primary care diagnostic work up of cognitive impairment using visual rating scales (VRS).

**Methods:**

Radiology reports were assessed regarding compliance with a contextual report template and the reporting of the parameters medial temporal lobe atrophy (MTA), white matter changes (WMC), global cortical atrophy (GCA), and width of lateral ventricles (WLV) using established VRS in two age-matched groups examined with NECT before (*n* = 111) and after (*n* = 125) the introduction of contextual reporting at our department. True positive rate (TPR) and true negative rate (TNR) before and after were compared.

**Results:**

We observed a significant increase in the percentage of radiology reports with mentioning of MTA from 29 to 76% (*p* < 0.001), WMC from 69 to 86% (*p* < 0.01), and GCA from 54 to 82% (*p* < 0.001). We observed a significant increase in the percentages of reports where all of the parameters were mentioned, from 6 to 29% (*p* < 0.001). There was a significant increase in TPR from 10 to 55% for MTA.

**Conclusion:**

This study suggests that contextual radiological assessment using VRS could increase the reporting frequency of radiology findings in the diagnostic work up of cognitive impairment but compliance with templates may be difficult to endorse.

**Key Points:**

*• Introducing visual rating scales in clinical practice increases the reporting frequency of MTA, WMC, and GCA in the diagnostic work up of subjective and mild cognitive impairment.*

*• Introducing visual rating scales has an effect on the true positive rate of reported MTA.*

*• Compliance with contextual radiology templates remains low when use of the template is not enforced by the department leadership.*

## Introduction

The work of the radiologist includes interpretation of images and communicating relevant findings through the radiology report. Traditionally, radiology reports have been free narratives with variations in structure and quality [1, 2]. The form of the radiology report has been debated and structured reporting has been suggested as a method to improve quality; however, consensus regarding form and style has not been reached [3–7].

Recently, contextual reporting has been suggested as an intermediate between structural reporting and free narrative reporting [8]. Contextual reports are structured in a disease-specific way with findings reported in a checklist manner but they are less strict compared with structural reporting. Another method to potentially improve quality is the use of established visual rating scales (VRS). An example from the field of neuroradiology is VRS developed for the investigation of cognitive impairment, which are endorsed in clinical practice [9–11].

Previous studies have shown that structural findings are underreported in the diagnostic work up of cognitive impairment [12–14]. A recent European survey showed that VRS were used in 75% of responding centers but structural reporting was used in only 28% [15]. In order to improve accuracy and clarity of radiology reports, our department introduced contextual reports as an endorsed routine. The purpose of this study is to investigate the effect on reporting of structural radiological findings after introducing a template with VRS in the primary care diagnostic work up of cognitive impairment.

## Materials and methods

### Materials

This is a retrospective, observational, single-center study. Eligible subjects, aged 60 to 80 years, were retrospectively recruited for two age-matched groups with exams performed before and after the introduction of contextual reporting; non-enhanced computed tomography (NECT) is the preferred modality in our country due to greater availability [16]. Only referrals issued by general physicians as part of a primary care diagnostic work up of cognitive impairment (the routine in our country) were eligible for inclusion.

We searched our picture archiving and communication system (PACS) for referrals containing the words “dementia” and/or “memory” together with the word “investigation” to identify eligible subjects under primary care investigation for subjective or mild cognitive impairment. Since our purpose was to study the effect of introducing VRS in the primary diagnostic work up, subjects where referrals mentioned known dementia or psychiatric disorder were not eligible for inclusion.

The group “before” was retrospectively recruited from the Swedish BioFINDER study mild cognitive impairment (MCI) cohort (see https://biofinder.se/ for more detail) where eligible subjects had performed a routine NECT available in our PACS from January 2010 to December 2014. Some subjects (*n* = 68) in this group have been included in a previous study but all results are new for this study [14]. The group “after” was retrospectively recruited from our PACS from January 2016 to December 2017. To mimic the clinical situation, we had only access to clinical information given in the referrals.

**Fig. 1:**
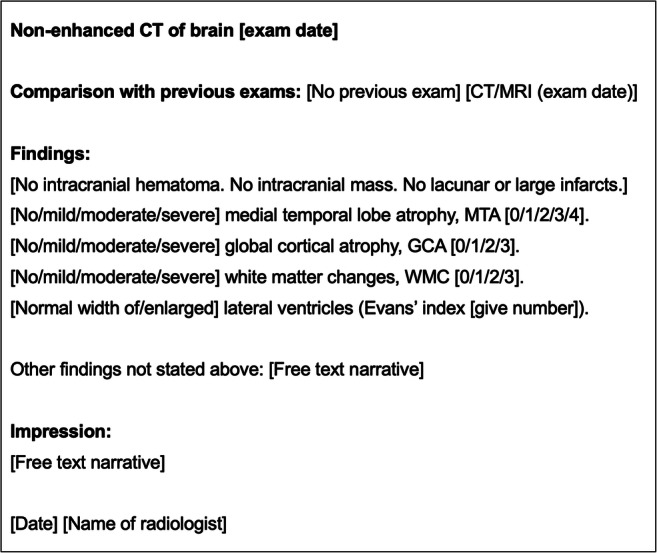
Contextual reporting template for the investigation of cognitive impairment. Free text narrative style can be used but evaluated parameters should always be mentioned regardless if they are normal or not. For full compliance, every report must include assessment of medial temporal lobe atrophy (MTA), global cortical atrophy (GCA), white matter changes (WMC), and with of lateral ventricles (WLV) and end with a separate “Impression”

Our endorsed template (see Fig. 1) states that medial temporal lobe atrophy (MTA), white matter changes (WMC), global cortical atrophy (GCA), and width of lateral ventricles (WLV) must be reported. The use of established VRS is endorsed [11]. All reports must end with an “Impression” where findings should be interpreted and probable diagnosis listed. For this study, any additional findings mentioned in the reports were not included in the evaluation. Full compliance was defined as reports including all evaluated parameters and an “Impression.”

### Assessment of exams

All NECT images were rated in a second reading by an experienced neuroradiologist (C.H.). Visual rating of MTA was done on coronal reformats with 3 mm slices (parallel to the axis of the brain stem) and graded from 0 = no atrophy to 4 = severe atrophy according to Scheltens’ MTA scale. Each side was rated separately and the overall highest score was given. Ratings were dichotomized into normal (0–1 if < 75 years, 0–2 if ≥ 75 years) and abnormal (2–4 if < 75 years, 3–4 if ≥ 75 years) [17, 18]. For rating of GCA, Pasquier’s scale was used on axial reformats in 5 mm slices (parallel to the orbitomeatal plane) and graded from 0 = no cortical atrophy to 3 = severe cortical atrophy [11, 19]. Grades were dichotomized into normal (0–1 regardless of age) and abnormal (2–3 regardless of age) based on previous studies [12, 14]. Fazekas’ scale was used to grade WMC with four grades from 0 = none or a single lesion to 3 = confluent lesions and dichotomized into normal (0 < 65 years, 0–1 ≥ 65 years) and abnormal (1–3 < 65 years, 2–3 ≥ 65 years) [11, 12, 20]. Evans’ index (EI) was measured on axial slices as an estimation of WLV. Cutoffs corrected for age and gender suggested by Brix et al were used (age: males/females); 65–69 years, 0.34/0.32; 70–74 years, 0.36/0.33; and 75–79 years, 0.37/0.34 [21, 22]. For 60–64 years, ≥ 0.30 was considered abnormal regardless of gender.

All NECT exams were performed according to our clinical routine with helical scan mode using Z-axis dose modulation on scanners from three different vendors with 120 kV voltage, exposure from 150 to 320 mAs, collimation from 0.5 to 0.75, and pitch factor from 0.36 to 0.65. Image quality was considered equal between scanners. All readings were done in our PACS IDS7® (Sectra AB) with a center width of 40 HU and window width of 80 HU.

### Assessment of clinical reports

All reports were reassessed and graded according to a scale by Torisson et al [12] and applied in accordance with a previous study [14] with respect to quantitative (e.g., “mild,” “severe”) and qualitative (e.g., “widened,” “enlarged”) descriptions of the evaluated parameters. Examples of how reports were graded are given in Table 1. Reports were graded as NA = not mentioned, 0 = normal (corresponds to MTA 0, GCA 0, and WMC 0), 1 = mild or reported but not quantified (corresponds to MTA 1–2, GCA 1, and WMC 1), 2 = moderate (corresponds to MTA 3, GCA 2, and WMC 2), and 3 = severe (corresponds to MTA 4, GCA 3, and WMC 3) [12]. For MTA, GCA, and WMC, a grade of ≥ 2 was considered abnormal. For WLV, a grade of ≥ 1 was considered abnormal. The gradings were compared with the second reading for estimation of true positive rate (TPR) and true negative rate (TNR). For this, we assumed that all reports where evaluated parameters had not been mentioned were assessed as normal. Since visual ratings are subjective, an additional rating was performed after 4 weeks for estimation of intra-rater agreement. Additionally, a second rater (D.v.W.) performed an assessment of GCA, MTA, and WMC on 100 randomly selected subjects (50 from each group) for estimation of inter-rater agreement and the rating with the highest inter-rater agreement was chosen as standard for the second reading.

**Table 1:** Examples from radiology reports and our quantification and grading in accordance with the scale by Torisson et al [12]

Examples from reports	Findings mentioned				Grading				Comment
	WLV	GCA	MTA	WMC	WLV	GCA	MTA	WMC	
“No abnormal changes”	No	No	No	No	NA	NA	NA	NA	No findings reported
“Normal attenuation of parenchyma. Normal CSF spaces. No atrophy”	Yes	Yes	No	Yes	0	0	NA	0	“Attenuation” interpreted as WMC. CSF spaces interpreted as WLV. MTA not mentioned
“No focal changes. Age adequate CSF spaces”	Yes	No	No	Yes	0	NA	NA	0	“Focal changes” interpreted as WMC. “Age adequate” interpreted as normal
“Mild WMC. Mild general atrophy. Increased width of ventricles”	Yes	Yes	No	Yes	1	1	NA	1	WLV mentioned but not graded. MTA not reported
“No ischemic lesion. Normal CSF spaces. No medial temporal lobe atrophy”	Yes	No	Yes	Yes	0	NA	0	0	“Ischemic lesion” interpreted as WMC. GCA not mentioned
“Increased width of lateral ventricles. Some atrophy. Medial temporal lobe atrophy, grade 3. Severe white matter changes”	Yes	Yes	Yes	Yes	1	1	2	3	ELV and GCA reported but not graded. MTA 3 interpreted as moderate

Scale by Torisson et al: *NA* = not mentioned in report, 0 = reported as normal, 1 = reported as mild or reported but not quantified, 2 = reported as moderate, 3 = reported as severe. *MTA* medial temporal lobe atrophy, *GCA* global cortical atrophy, *WMC* white matter changes, *WLV* with of lateral ventricles, *CSF spaces* cerebrospinal fluid spaces

### Statistics

Descriptive statistics (percentages) were estimated to summarize results of the gradings and the second reading. True positive rate and TNR were calculated using MEDCALC® (MedCalc Software Ltd.) online statistics calculator (available at https://www.medcalc.org/calc/diagnostic_test.php) where 95% confidence intervals (95% CI) were visually compared for statistical significance. Difference between groups was compared using Pearson chi-square analysis and Mann-Whitney *U* test where applicable. For estimation of intra- and inter-rater agreement, Cohen’s *κ* was estimated for dichotomized data. The level of agreement was defined according to Landis and Koch [23]. Calculations were done using SPSS® version 26 (IBM Corporation). A *p* < 0.05 was considered statistically significant.

## Results

We identified 251 eligible subjects, ten subjects had cancelled examinations, four subjects had failed to perform the exam, and the referral was unclear for one subject. These were excluded and 236 subjects were included with 111 examined “before” and 125 “after” the introduction of contextual reporting. There were no significant differences between the groups with respect to prevalence of abnormal findings and gender. Subjective cognitive impairment was the reported symptom in 97% of all subjects (see Table 2 for demographic data). Evans’ index was only reported for one subject and was included in WLV.

**Table 2:** Data on subjects and prevalence of evaluated parameters for the two groups “before” and “after”

	“before”	“after”	*p* value
Subjects			
Number of subjects (n)	111	125	-
Age in years (median, interquartile range)	72 (7.8)	73 (7.3)	0.02*
Females	47%	49%	0.76
Clinical symptom (as reported in referrals)			
Subjective cognitive impairment	95%	98%	0.19
Personality changes	7%	2%	*0.03*
Confusion	1%	2%	0.63
Prevalence (abnormal in the second reading)			
Abnormal MTA	18%	18%	0.93
Abnormal WMC	25%	22%	0.51
Abnormal GCA	17%	22%	0.39
Abnormal WLV	17%	15%	0.69

Significant differences are italicized. Pearson chi-square except ^*^Mann-Whitney *U* test; *p* < 0.05 represents a significant difference. *MTA* medial temporal lobe atrophy, *GCA* global cortical atrophy, *WMC* white matter changes, *WLV* with of lateral ventricles

Intra-rater agreement was excellent for MTA *κ* = 0.82 95% CI (0.72 to 0.91), *p* < 0.001 and WLV *κ* = 0.87 95% CI (0.78 to 0.96), *p* < 0.001; substantial for WMC *κ* = 0.79 95% CI (0.70 to 0.88), *p* < 0.001; and moderate for GCA *κ* = 0.57 95% CI (0.44 to 0.71), *p* < 0.001. The highest inter-rater agreement for 100 randomly selected subjects showed substantial agreement for MTA *κ* = 0.73 95% CI (0.55 to 0.92), *p* < 0.001; excellent agreement for WMC *κ* = 0.81 95% CI (0.69 to 0.94), *p* < 0.001; and fair agreement for GCA *κ* = 0.44 95% CI (0.19 to 0.70), *p* < 0.001.

Data on grading of clinical reports and concordance with our second reading are summarized in Table 3. In total, MTA was reported in 54% of the reports. The corresponding number for WMC, GCA, and WLV was 78%, 69%, and 59% respectively. Where MTA was reported as moderate to severe (i.e., Torisson’s scale grades 2–3), 88% was correctly reported as abnormal compared with the second reading. Medial temporal lobe atrophy was reported as mild (i.e., grade 1), in 18% of the reports. The corresponding number in the second reading was 45%; when age correction was applied, 36% was normal and 9% abnormal of which 0% (*n* = 0) was correctly reported as abnormal. Where WMC was reported as moderate to severe, 83% was correctly reported as abnormal compared with the second reading. White matter changes were reported as mild in 31% of the reports; the corresponding number in the second reading was 18%; when age correction was applied, 17% was normal and 1% abnormal of which 0% (*n* = 0) was correctly reported as abnormal. Where GCA was reported as moderate to severe, 47% was correctly reported as abnormal, and for WLV, the figure was 38% compared with the second reading.

**Table 3:** Grading of clinical reports and concordance with second reading

	Clinical report	Second reading	Abnormal in second reading	Normal in second reading	Correctly reported as abnormal in clinical report	Total abnormal in second reading
MTA						18%
“0”	29%	47%	--	47%	--	
“1”	18%	45%*	9%^1^	36%^1^	0%	
“2”	5%	6%	6%	--	4%	
“3”	2%	2%	2%	--	2%	
“NA”	46%	--	--	--	--	
WMC						23%
“0”	27%	60%	--	60%	--	
“1”	31%	18%	1%^2^	17%^2^	0%	
“2”	10%	9%	9%	--	7%	
“3”	10%	13%	13%	--	10%	
“NA”	22%	--	--	--	--	
GCA						19%
“0”	31%	27%	--	27%	--	
“1”	30%	54%	--	54%	--	
“2”	7%	18%	18%	--	3%	
“3”	< 1%	1%	1%	--	< 1%	
“NA”	31%	--	--	--	--	
WLV						16%
“0”	39%	84%	--	84%	--	
“1”+“2”+“3”^5^	20%	16%	16%	--	8%	
“NA”	41%	--	--	--	--	

All values are rounded to the nearest integer and represents percentages of total (*N* = 236) study population. *Sum of MTA 1 (27%) and MTA 2 (18%) in the second reading. ^1^Corrected for age where MTA 2 is abnormal if age < 75 years. ^2^Corrected for age where WMC 1 is abnormal if age < 65 years. ^5^Sum of “1” + “2” + “3,” enlarged WLV dichotomized according to age-corrected cutoffs suggested by Brix et al [22]. The scale by Torisson et al [12]: *NA* = not mentioned in report, 0 = reported as normal, 1 = reported as mild or reported but not quantified, 2 = reported as moderate, 3 = reported as severe. *MTA* medial temporal lobe atrophy, *GCA* global cortical atrophy, *WMC* white matter changes, *WLV* width of lateral ventricles

Data on frequencies and compliance, including differences between the groups, are summarized in Table 4. Reporting of MTA, WMC, and GCA increased significantly. There was no significant change in the reporting of WLV. Altogether, the percentage of reports with all parameters mentioned increased from 6% (*n* = 7) to 29% (*n* = 36). Full compliance remained low; the percentage of reports in strict full compliance with the template increased from 2% (*n* = 2) to 8% (*n* = 10).

**Table 4:** Percentage of original reports mentioning the evaluated parameters outlined in the contextual reporting template and compliance with contextual reporting for the two groups “before” and “after”

	“before” (*n* = 111)	“after” (*n* = 125)	p*
Evaluated parameter	Percentage of original reports with parameters mentioned		
MTA	29%	76%	*< 0.001*
WMC	69%	86%	*< 0.01*
GCA	54%	82%	*< 0.001*
WLV	55%	62%	0.25
	Percentage of original reports with all parameters mentioned		
All parameters reported	6%	29%	*< 0.001*

*Pearson chi-square, *p* < 0.05 represents a significant increase in percentage of reports with the evaluated parameter mentioned, significant changes are italicized. *MTA* medial temporal lobe atrophy, *GCA* global cortical atrophy, *WMC* white matter changes, *WLV* with of lateral ventricles

Results regarding TPR and TNR are summarized in Table 5. A significant increase in TPR was observed for MTA with an increase from 10 to 55%. There were no significant changes in TPR for the other parameters but an increase from 0 to 33% was observed for GCA and an increase from 37 to 58% was observed for WLV. There was high to almost perfect TNR with no significant changes for any parameter in the two groups.

**Table 5:** True positive rate and true negative rate for the evaluated parameters, expressed as percentages, of original reports in the two groups “before” and “after”

	“before” (*n* = 111)				“after” (*n* = 125)			
Evaluated parameter	MTA	WMC	GCA	WLV	MTA	WMC	GCA	WLV
TPR % (95% CI)	10 (1–32)	68 (48–84)	0 (0–18)	37 (16–62)	*55 (32–76)*	78 (58–91)	33 (17–54)	58 (34–80)
TNR % (95% CI)	99 (94–100)	99 (93–100)	96 (89–99)	88 (80–94)	99 (95–100)	93 (86–97)	94 (87–98)	82 (73–89)

Significant changes are italicized. *MTA* medial temporal lobe atrophy, *GCA* global cortical atrophy, *WMC* white matter changes, *WLV* width of lateral ventricles, *95% CI* 95% confidence interval, *TPR* true positive rate, *TNR* true negative rate

## Discussion

In this retrospective, observational study, we evaluated compliance and compared TPR of radiology reports before and after the introduction of contextual reporting in the diagnostic work up of cognitive impairment. We found an increase in the reporting of MTA, GCA, and WMC and an increased TPR for MTA. Although an increase in the reporting of evaluated parameters was observed, full compliance with the template remained low (8%) and the percentage of reports where all parameters were mentioned only reached 29%. Due to small numbers, it is difficult to draw any definitive conclusions from the reports with full compliance or mentioning of all parameters why we chose to evaluate each parameter separately.

We had anticipated that full compliance would reach at least 50% why our results would seem disappointing. In a study by Powell et al, 9% compliance was observed when a structured template for assessing maxillofacial trauma was evaluated [24]. This figure is close to our result but differences in methodology make further comparisons difficult. Another study by Larson et al showed that structured reporting could successfully be implemented if enforced by the department leadership [4]. Since visual ratings are subjective, it has also been suggested that differences in structure of radiology reports may be explained by local traditions and, in the case of cognitive impairment, imaging has traditionally been used to exclude secondary causes to cognitive impairment [25, 26]. Taking all of this into consideration, we believe our observed low compliance is similar to what have been previously reported and could be explained by adherence to local traditions. Also, the use of our template was not enforced by the department leadership.

Previous studies have shown that abnormal findings, in particular MTA, are underreported in radiology reports even when assessment is warranted [12, 14]. Medial temporal lobe atrophy is an important structural finding in Alzheimer’s disease (AD) but it can also be found in other dementias [27, 28]. Our results showed an increase in reporting and TPR for MTA. Moderate to severe MTA was correctly reported as abnormal in 88% but mild atrophy was underreported; also, when age correction was applied, there remained an underreporting of abnormal mild atrophy (i.e., MTA 2 in subjects < 75 years). The underreporting of mild MTA probably explains the observed low TPR (10% “before” and 55% “after”) for MTA and it cannot be excluded that a study population with a higher prevalence of moderate to severe MTA would have resulted in better TPR and compliance. In line with previous studies, excellent intra-rater and substantial inter-rater agreement was observed for MTA [14, 29]. With respect to the clinical importance of MTA and previously observed underreporting, we believe our results regarding MTA have an important clinical impact [12, 14].

The reporting of GCA increased significantly, although TPR remained low (33%). Where abnormal GCA was reported, it was erroneous in 53% which probably explains our observed low TPR. The GCA scale covers a larger brain region compared with the MTA scale. Although ratings are based on the highest grade of atrophy, the risk of potential underreporting cannot be eliminated since moderate parietal cortical atrophy and mild frontal cortical atrophy still could be interpreted as overall mild GCA (normal) by one rater and moderate GCA (abnormal) by another. This would probably also explain our observed levels of agreement.

There was an increase in the reporting of WMC and WLV, where the increase for WMC was significant, but changes in TPR were not significant. In many reports, the phrase “normal appearing cerebrospinal fluid spaces” (CSF spaces) was used. This resulted in difficulties in our grading of the reports since this could mean normal width of sulci (i.e., normal GCA) and normal WLV combined. We chose to interpret this as normal WLV. White matter changes are preferably assessed on magnetic resonance imaging (MRI), but for abnormal findings, NECT is considered sufficient [9, 11]. In other words, when WMC is reported on NECT, it is most likely to be Fazekas grade 2 or 3. Our results showed that moderate to severe WMC was reported in 20% of the reports compared with 22% in the second reading while mild changes were overreported. In most reports, the phrases “white matter changes” or “focal parenchymal changes” were used to describe WMC which posed no difficulties in our grading why we do not believe this explains our results. Radiologists have been shown to be keener to report WMC which we believe is a more probable explanation to our results [14]. Our results would suggest that contextual reporting only had a significant effect on the reporting of MTA but the increased reporting of the other parameters would suggest that discrepancies in reporting styles were reduced to some extent. However, the low compliance with our contextual template and our assumption that reports with no mentioning of the evaluated parameters were normal may hamper such conclusions.

There are limitations to this study: (i) The use of two different cohorts could result in a potential bias from cohort effects. Differences in prevalence of abnormal findings were not significant between the groups but the observed prevalence was probably lower than would be expected in a memory clinic population. Our study population was derived from a population with cognitive impairment where none was diagnosed with dementia since we believe the clinical benefit from using VRS would be greater in this group [30, 31]. It cannot be excluded that an older study population or a memory clinic population would have yielded a higher prevalence of abnormal findings where a different compliance with our template cannot be excluded. (ii) Visual ratings are subjective and quantitative data such as volume segmentation would be preferable but are difficult to perform with NECT. We chose a gold standard based on high inter-rater agreement but this approach does not exclude a potential rater bias. (iii) The retrospective design hampers the possibility to obtain reliable data on potential effects of training, education, or individual experiences of using VRS among the neuroradiologists at our department. (iv) We have not compared our template with other structural reporting templates and we have not followed up on how the use of VRS affects the final diagnosis. (v) The use of the scale suggested by Torisson et al can be questioned. This is an attempt to grade the qualitative data in the radiology reports to make comparisons possible but the scale has not been tested for rater reliability, although it has been used in previous studies [12, 14].

This study adds knowledge to how reporting frequency of radiology findings can be improved in the diagnostic work up of cognitive impairment. Our results suggest that there is a possibility to increase the overall reporting of structural findings but only the results for MTA were significant. In conclusion, this study suggests that contextual radiological assessment using VRS could increase the reporting frequency of radiology findings in the diagnostic work up of cognitive impairment, but compliance with templates may be difficult to endorse.

## Abbreviations

AD: Alzheimer’s disease
BioFINDER: Biomarkers for identifying neurodegenerative disorders early and reliably
CI: Confidence interval
CSF spaces: Cerebrospinal fluid spaces
CT: Computed tomography
EI: Evans’ index
GCA: Global cortical atrophy
HU: Hounsfield units
MRI: Magnetic resonance imaging
MTA: Medial temporal lobe atrophy
NECT: Non-enhanced computed tomography
PACS: Picture archiving and communication system
TNR: True negative rate
TPR: True positive rate
VRS: Visual rating scales
WLV: Width of lateral ventricles
WMC: White matter changes
